# Efficacy of Lactic Acid and Modified Atmosphere Packaging against *Campylobacter jejuni* on Chicken during Refrigerated Storage

**DOI:** 10.3390/foods9010109

**Published:** 2020-01-20

**Authors:** Elena Gonzalez-Fandos, Naiara Maya, Alba Martínez-Laorden, Iratxe Perez-Arnedo

**Affiliations:** Food Technology Department, CIVA Research Center, University of La Rioja, 26006 La Rioja, Spain; naiara.maya@unirioja.es (N.M.); alba.martinezl@unirioja.es (A.M.-L.); ipearn@gmail.com (I.P.-A.)

**Keywords:** food safety, *Campylobacter*, poultry, organic acids, decontamination, foodborne pathogens

## Abstract

The present study was conducted to evaluate the combined effect of lactic acid washing and modified atmospheres packaging on the counts of *Campylobacter jejuni* on chicken legs stored at 4 °C. In experiment 1, inoculated chicken legs were washed with either 1% or 2% lactic acid solution for 5 min or distilled water (control). The treatment with 2% lactic acid reduced *C. jejuni* counts 1.42 log units after treatment (day 0). In experiment 2, inoculated samples were packaged under different conditions: air, 100%N_2_, vacuum, 20%CO_2_/80%N_2_, or 40%CO_2_/60%N_2_. *C. jejuni* counts were higher in samples packaged under vacuum or atmospheres containing CO_2_ than in air. In experiment 3, inoculated chicken legs were washed with a 2% lactic acid solution for 5 min or distilled water (control). Samples were packaged under different conditions: air, vacuum, 20%CO_2_/80%N_2_, or 40%CO_2_/60%N_2_. *C. jejuni* counts were lower in samples treated with lactic acid than in samples non-treated. However, *C. jejuni* counts were higher in chicken legs treated with lactic acid and packaged in modified atmospheres than in those treated and packaged in air. Immersion of chicken legs in a solution containing 2% lactic acid can reduce *C. jejuni* counts on fresh chicken packaged in modified atmosphere.

## 1. Introduction

Campylobacteriosis is one of the most frequently reported foodborne illness in the European Union, with 246,158 confirmed human cases in 2017, and an incidence rate of 64.8 cases per 100,000 population [1]. The prevalence of *Campylobacter* spp. in chicken meat is high [1,2,3]. In fact, chicken meat is considered as the main foodborne source of human campylobacteriosis [4].

Risk assessments for *Campylobacter* have concluded that a 2 log reduction of the counts of *Campylobacter* on chicken meat will lead to a 30-fold reduction of human campylobacteriosis cases associated with chicken meat [5]. Organic acids are generally recognized as safe substances (GRAS), being traditionally used as food preservatives [6]. In order to reduce *Campylobacter* spp. counts on chicken meat the treatment with organic acids could be useful [7,8]. The effectiveness of organic acids for reducing pathogens depends on the acid type, acid concentration, temperature, contact time, tissue type, or organisms [9].

Several research reports have addressed the efficacy of lactic acid for reducing microbial counts in meat and poultry [9,10]. Treatments with lactic acid at varying concentrations result in microbial reductions ranging from 1 to 3 log units on meat surfaces [10]. Decontamination of beef carcasses with solutions of lactic acid at concentrations between 2% and 5% has been permitted in the European Union since 2013 [11]. Recently, the safety and efficacy of lactic acid to reduce microbial contamination on pork have been evaluated by the European Food Safety Authority (EFSA) [12]. EFSA has concluded that the treatments using lactic acid for decontamination pork are of no safety concern, provided that the substance used complies with Union specifications for food additives and are effective to reduce microbiological surface contamination [12]. The decontamination of poultry carcasses with lactic acid is currently not yet allowed for poultry meat in the European Union.

Different research reports have addressed the use of lactic acid for reducing *Salmonella* [13] and *Listeria monocytogenes* [14]. The ability of lactic acid to inhibit *Campylobacter jejuni* has been studied in laboratory media [15,16,17] and meat [18,19].

Chicken meat is a very perishable food, packaging in modified atmosphere can extend its shelf life, since aerobic spoilage bacteria such as *Pseudomonas* are inhibited [20]. Nevertheless, there is a great concern about the microbiological safety of modified atmosphere packaging (MAP) products, since some pathogens are able to grow before spoilage becomes evident. As a microaerophilic and capnophilic microorganism, *C. jejuni* requires an atmosphere with reduced oxygen and elevated carbon dioxide concentration for its growth [21]. The effect of different modified atmospheres packaging on the survival of *C. jejuni* has been studied by Meredith et al. [22]. Some studies evaluate the survival of *C. jejuni* in culture media treated with lactic acid and incubation under different atmospheres [23]. However, there are few studies on the combined effect of lactic acid and modified atmospheres against *C. jejuni* in chicken meat [24].

The aim of this work was to evaluate the effectiveness of lactic acid washing to reduce *C. jejuni* on chicken meat packaged under modified atmospheres and stored at 4 °C. Microbiological and sensorial quality were also evaluated.

## 2. Materials and Methods

### 2.1. Preparation of Bacterial Inoculum

*C. jejuni* ATTCC 33291 was grown in Preston Campylobacter enrichment broth (Oxoid, Hampshire, UK) under microaerobic conditions at 42 °C for 24 h. The inoculum was prepared according to the methodology described by Gonzalez-Fandos and Maya [8]. *Campylobacter jejuni* cells were diluted in sterile peptone water to yield a concentration of about 10^6^ cfu/mL.

### 2.2. Inoculation of Poultry

Fresh chicken legs were collected from a poultry processing plant immediately after chilling (La Rioja, Spain). The legs were placed on crushed ice and transported to the laboratory. The chicken legs were inoculated with *C. jejuni* by dipping them into a suspension of this pathogen for 5 min at room temperature. After inoculation, the legs were removed and kept for 30 min at room temperature to allow the bacteria attachment.

### 2.3. Experiment 1. Treatment with Lactic Acid

Inoculated legs were divided into three groups, each containing 15 legs. Samples of each group were immersed for 5 min into the following solutions: 1% lactic acid, 2% lactic acid, or distilled water (control). Lactic acid was provided by Scharlau (Barcelona, Spain). After treatment, the legs were removed and drained for 5 min at room temperature. Afterwards, legs were placed individually in sterile bags and stored at 4 °C for 8 days. Samples were taken on days 0 (after treatment), 1, 3, 6, and 8. On the sampling days, three legs of each group were taken out of storage to perform microbiological, pH, and sensorial analysis.

Since better results were obtained in samples washed with 2% lactic acid, these washing conditions were selected in the experiment 3 carried out with modified atmosphere packaging.

### 2.4. Experiment 2. Packaging in Modified Atmosphere

After inoculation chicken legs were divided into five groups, each containing 24 legs. Group 1 samples were used as the control and were packaged in air (Batch AC). Legs from Groups 2–5 were placed in plastic bags provided by Dixie (Dixie, Bern, Switzerland) and packaged under different modified atmospheres: 100%N_2_, vacuum, 20%CO_2_/80%N_2_, and 40%CO_2_/60%N_2_.

The characteristic of the plastic film used were as follows: CO_2_ permeability less than 13 cm^3^/m^2^/24 h/atm at 25 °C, O_2_ permeability less than 5 cm^3^/m^2^/24 h/atm, water vapor transmission rate less than 1.8 g/m^2^/24 h. A Vaessen-Schoemake machine was used for packaging (Vaessen-Schoemake, Barcelona, Spain). The gases used were provided by Praxair (Madrid, Spain). After packaging all the samples were stored at 4 °C for 15 days. Samples were analyzed on day 0 and after 1, 3, 6, 8, 10, 13, and 15 days of storage. On the sampling days, three legs of each group were removed of storage to determine gases concentration and perform microbiological, sensorial, and pH analysis.

### 2.5. Experiment 3. Combined Treatment of Lactic Acid and Modified Atmosphere Packaging

Inoculated legs were divided into two groups. Samples from Group 1 containing 120 legs were immersed for 5 min into a solution containing 2% lactic acid, samples from Group 2 containing 30 legs were immersed in the same conditions on distilled water (control). After treatment, the legs were removed and drained for 5 min: legs from Group 1 were packaged under different modified atmospheres as described in experiment 2. The modified atmospheres selected in this experiment were 20%CO_2_/80%N_2_, 40%CO_2_/60%N_2_, and vacuum. Legs from Group 2 were packaged in air (Batch BC, control). Samples were taken on days 0 (after treatment), 1, 3, 6, 8, 10, 13, 15, and 17. On the sampling days, three legs of each group were taken out of storage to perform microbiological, pH, and sensorial analysis.

### 2.6. Microbiological Analyses and pH Determination

Ten grams of skin were aseptically weighed and homogenized in a Stomacher (IUL, Barcelona, Spain) for 2 min with 90 mL of 1% sterile peptone water (Oxoid). Decimal dilutions were made with the same diluent. Mesophilic microorganisms were determined on plate count agar (PCA, Merck, Darmstadt, Germany) using the pour plate method, incubating at 30 °C for 72 h [25]. Psychrotrophic bacteria were determined on PCA (Merck) with an incubation temperature of 7 °C for 10 days, using the pour plate method [25]. *Pseudomonas* spp. were determined on King´s B medium (Merck) with an incubation temperature of 25 °C for 48 h. [8]. *Enterobacterales* were determined on violet red bile glucose (VRBG) (Merck) following the pour plate method with an incubation temperature of 37 °C for 48 h [25,26]. Enumeration of *C. jejuni* was carried out on modified Charcoal cefoperazone desoxycolate agar (Oxoid) with an incubation temperature of 42 °C for 48 h under microaerobic conditions according to ISO 10272-2 [27]. Confirmation of five presumptive colonies was performed according to the ISO 10272-2 principles [27]. Measurements of pH were made using a Crison model 2002 pH meter with a penetration electrode (Crison Instruments, Barcelona, Spain).

### 2.7. Sensorial Analysis

The samples were evaluated for overall acceptability with regard to odor, color, and overall appearance by a panel of nine members as described by González-Fandos and Maya [8]. A structured hedonic scale with numerical scores ranging from 7 to 1 was used. A score of 3 was considered the borderline of acceptability.

### 2.8. Statistical Analysis

Analysis of variance was performed using the SYSTAT program for Windows; Statistics version 5.0 (Evanston, IL, USA, 1992). For assessing the data were normally distributed the Shapiro–Wilk test was carried out. The homogeneity of variance was evaluated according to the Bartlett’s test. Tukey’s test for comparison of means was performed using the same program. Plate count data were converted to logarithms prior to their statistical treatment. Significance level was defined at *p* < 0.05.

## 3. Results

### 3.1. Effect of Lactic Acid

The effect of lactic acid washing on mesophilic, psychrotrophic, *Pseudomonas, Enterobacterales*, and *C. jejuni* counts in chicken legs is shown in Table 1. Significant differences (*p* < 0.05) in mesophile, psychrotroph, and *Pseudomonas* counts were observed between the legs treated with lactic acid and the control legs. The results obtained showed that washing with 2% lactic acid reduced psychrotrophs counts between 1.31 and 2.07 log units compared to the control legs throughout storage. Significant differences (*p* < 0.05) in psychrotrophs counts were observed between the legs treated with 1% lactic acid and those treated with 2% lactic acid on days 6 and 8 of storage. Significant differences (*p* < 0.05) in *Enterobacterales* counts were observed between the legs treated with lactic acid and the control legs. The results obtained showed that washing with 2% lactic acid reduced *Enterobacterales* counts between 0.5 and 1.94 log units compared to the control legs throughout storage. No significant differences (*p* > 0.05) in *Enterobacterales* counts were observed between the legs treated with 1% lactic acid and those treated with 2% lactic acid, except on day 1 of storage, although lower counts were observed in chicken legs treated with 2% lactic acid.

Significant differences (*p* < 0.05) in the *C. jejuni* counts were observed on legs treated with 2% lactic acid compared to the control samples. After 1 day of storage, *C. jejuni* counts were 1.5 log units lower in legs treated with 2% lactic acid than in control ones. Significant reductions (*p* < 0.05) in the *C. jejuni* counts were also observed on legs treated with 1% lactic acid on days 0, 1, and 3 of storage compared to the control samples. However, no significant differences were observed on days 6 and 8 of storage.

Lactic acid treatment significantly reduced (*p* < 0.05) the pH of legs. Initial pH values in chicken legs treated with 1% or 2% lactic acid were 5.83 ± 0.15 and 4.96 ± 0.17, respectively (0.6 and 1.47 units lower than in control samples). Significant differences (*p* < 0.05) in pH values between legs treated with 1% lactic and those treated with 2% lactic acid were only observed on day 0 (after treatment). On day 8, pH values were 6.80 ± 0.29, 6.04 ± 0.04, and 5.91 ± 0.38 in control legs, legs treated with 1% and 2% lactic acid, respectively. Lactic acid at concentrations of 1% and 2% did not negatively affect the sensorial quality since scores of 7 in overall acceptability were observed after treatment (Table 2). Control legs were rejected on day 6 of storage, while those treated with 2% lactic acid remained acceptable until day 8 of storage. Chicken legs treated with 1% lactic acid were rejected on day 8 of storage. Since better results were obtained in samples washed with 2% lactic acid, these conditions were selected in the study carried out with modified atmosphere packaging.

### 3.2. Effect of Modified Atmosphere Packaging

Table 3 shows the effect of packaging in modified atmospheres on mesophilic, psychrotrophic, *Pseudomonas, Enterobacterales*, and *C. jejuni* counts in chicken legs. Significant differences (*p* < 0.05) in mesophilic, psychrotrophic, and *Pseudomonas* counts were observed between the legs packaged in vacuum or atmospheres containing CO_2_ (20%CO_2_/80%N_2_ and 40%CO_2_/60%N_2_) and the control legs packaged in air, except on day 0. Packaging in 40%CO_2_/60%N_2_ (Batch AC40) reduced psychrotrophic counts between 1.24 and 2.12 log units compared to the control legs (Batch AC) throughout storage. Only significant differences (*p* < 0.05) in *Enterobacterales* counts were observed between the control legs packaged in air and the legs packaged in atmospheres containing 40% CO_2_ except on day 0 and the legs packaged in atmospheres containing 20% CO_2_ on day 1 of storage.

Significant differences (*p* < 0.05) in the *C. jejuni* counts were observed on legs packaged in modified atmosphere (Batches AN, AV, AC20, and AC40) and control samples (Batch AC) after 3 days of storage, being the counts of this pathogen higher in the samples packaged in MAP.

Significant differences (*p* < 0.05) were found in pH values between samples packaged in modified atmospheres containing CO_2_ and those packaged in air, vacuum, or 100%N_2_, except on day 0. On day 1 pH on control samples was 6.45 ± 0.16, while in samples packaged in 40%CO_2_/60%N_2_ was 5.80 ± 0.05. Changes in packages atmospheres (CO_2_, O_2_, N_2_) exhibited no significant differences (*p* > 0.05) during storage among samples packaged in the same atmosphere condition. Slight changes in CO_2_ concentrations in the pack atmospheres were detected in Batches AC20 and AC40. CO_2_ concentrations decreased by about 4% during storage in MAP samples. The sensorial acceptability of chicken legs was not adversely affected by the packaging conditions, receiving higher scores than control samples after 1 day of storage (Table 4). Control legs were rejected on day 6, while those packaged in 40%CO_2_/60%N_2_ (Batch AC40) and 20%CO_2_/80%N_2_ (Batch AC20) were rejected on days 15 and 13, respectively. Samples packaged in 100%N_2_ were rejected on day 8, while those packaged under vacuum were rejected on day 13 of storage.

### 3.3. Combined Treatment of Lactic Acid and Modified Atmosphere Packaging

Table 5 shows the effect of 2% lactic acid on mesophile, psychrotroph, *Pseudomonas*, *Enterobacterales*, and *C. jejuni* counts on chicken legs packaged in different modified atmospheres. Significant differences (*p* < 0.05) in mesophiles, psychrotroph, and *Pseudomonas* counts were found between the legs treated with 2% lactic packaged in air (Batch BA) and those packaged in atmospheres containing CO_2_ (Batch BC20 and BC40) throughout storage. Significant differences (*p* < 0.05) in psychrotroph and *Pseudomonas* counts were found between the legs treated with 2% lactic packaged in air (Batch BA) and those packaged in vacuum after day 6 of storage, although lower counts were observed in those packaged in vacuum throughout storage. The air-packaged legs non-treated with lactic acid had the fastest increase in psychrotroph and *Pseudomonas* counts (Batch BC). The lowest psychrotroph and *Pseudomonas* counts were observed in those samples treated with lactic acid and packaged in 40%CO_2_/60%N_2_ (Batch BC40), with psychrotroph reductions counts between 2.02 and 4.74 log units compared to the control legs non-treated with lactic acid and packaged in air (Batch BC) throughout storage. Significant differences (*p* < 0.05) in *Enterobacterales* counts were found between the legs treated with 2% lactic packaged in air (Batch BA) and those packaged in atmospheres containing CO_2_ (Batch BC20 and BC40) or vacuum after day 3 of storage.

Significant differences (*p* < 0.05) in the *C. jejuni* counts were observed on legs treated with 2% lactic acid (Batch BA, BV, BC20, and BC40) compared to the control samples non-treated with lactic acid (Batch BC). No significant reductions (*p* > 0.05) were found in the *C. jejuni* counts among legs treated with 2% lactic acid and packaged in air, 20%CO_2_/80%N_2_, 40%CO_2_/60%N_2_, or vacuum, except on day 13 of storage, although the counts were higher in those samples packaged in atmospheres containing CO_2_ than in air or vacuum conditions. On day 3 samples washed with 2% lactic acid and packaged in an atmosphere containing 40%CO_2_/60%N_2_ showed a reduction of *C. jejuni* counts about 1.41 log units compared with poultry legs non-treated and packaged in air.

Initial pH values in legs treated with solutions containing 2% lactic (day 0) were 4.93 ± 0.02, 1.5 units lower than in control legs (Batch BC). Significant differences (*p* < 0.05) were found in pH values between samples washed with 2% lactic acid and packed in atmospheres containing CO_2_ and those packed in air or vacuum after 3 days of storage. Changes in packages atmospheres (CO_2_, O_2_, N_2_) exhibited no significant differences (*p* > 0.05) during storage among samples packaged in the same atmosphere condition. Slight changes in CO_2_ concentrations in the pack atmospheres were detected in Batches BC20 and BC40. CO_2_ concentrations decreased by about 4% during storage in MAP samples. Legs non-treated with lactic acid and packaged in air were rejected on day 6 (Batch BC), while those treated with lactic acid and packaged in 40%CO_2_/60%N_2_ remain acceptable until day 17 (Table 6). Chicken legs treated with lactic acid and packaged in 20%CO_2_/80%N_2_ were rejected on day 17. The use of modified atmosphere packaging in combination with lactic acid resulted in a shelf life extension of refrigerated fresh chicken legs.

## 4. Discussion

In the present work it was observed that washing with 1% or 2% lactic acid caused significant reductions (*p* < 0.05) in mesophilic, psychrotrophic, *Pseudomonas*, and *Enterobacterales* counts compared to untreated samples. These results agree with those reported by other authors as can be observed in Table 7 [28,29,30,31]. The differences found among the studies could be explained since longer treatments were applied by some authors (10 min instead of 5 min). In the present work, all the samples were dipped in a suspension with *C. jejuni*. Therefore, the cell counts of mesophliles, psychrotrophs, *Pseudomonas*, and *Enterobacterales* could be biased by the treatment.

A decrease in *C. jejuni c*ounts were observed in all the samples stored at 4 °C, other authors have also observed this decrease under refrigeration storage [32]. *C. jejuni* is a thermophilic bacterium and it is not able to grow under refrigeration [33]. Moreover, as a microaerophilic and capnophilic microorganism, *Campylobacter jejuni* requires an atmosphere with reduced oxygen and elevated carbon dioxide concentration for its growth [21]. Moreover, *Campylobater* spp. survive better in CO_2_ than in air conditions [22]. According to the results of the present study washing in lactic acid caused a significant reduction (*p* < 0.05) of *C. jejuni* counts compared to untreated legs. Baserisali et al. [15] observed that in culture media lactic acid is more effective against *C. jejuni* than other organic acids such as citric or propionic. The efficacy of lactic acid to inhibit *C. jejuni* appears to be higher in laboratory media than in chicken meat according to the results reported by other authors [17]. Chicken meat may have a protective effect due to their buffering capacity [34]. This fact could explain the lower effect against *C. jejuni* of lactic acid on chicken meat compared to the effect in broth. In contrast, Zhao and Doyle [16] reported that a treatment with 1% lactic acid did not reduce substantially *C. jejuni* counts in broth. Other authors have also reported that the treatment of chicken meat with lactic acid was effective for reducing *C. jejuni* counts as can be observed in Table 7 [19,32,34,35]. The higher reductions observed on chicken skin than in breast could be explained by the buffer capacity of chicken breast, thus pH values of breast treated with lactic acid were higher than those of treated chicken skin. The higher reduction observed in breasts compared to legs could be explained by the lower pH in breast compared to leg meat [36]. The antimicrobial effect of lactic acid is correlated with the level of undissociated acid form. The lower the pH, the higher level of undissociated organic form that can passively cross the cell membrane and enter the cell [37]. On other hand, lactic acid has a low molecular weight (90.08 Da) and is water soluble, thus this organic acid can easily penetrate the membrane of bacteria and modify its integrity [38]. The differences in efficacy of lactic acid on *C. jejuni* found in previous studies can be explained by variations in the portion of chicken meat (legs, breast, wings), concentration of lactic acid used, exposure times, temperature, application method (spray, immersion), inoculum size, and strains of *C. jejuni*.

The shelf life of chicken legs was extended in those samples treated with lactic acid. Mesophilic and psychrotrophic counts remained below 8 log cfu/g in legs treated with 2% lactic acid, while counts above 8 log cfu/g were reached in control samples on day 6 of storage. These results agree with those reported by other authors [28,31]. As Kolsarici and Candongan [28] we did not observe any adverse effect on sensory quality in chicken meat treated with 2% lactic acid. As in the present work Van der Marel et al. [30] observed that lactic acid treatments caused a decline in the pH of the poultry meat depending on the lactic acid concentration used.

Additionally, Meredith et al. [22] observed that packaging in 30%CO_2_/70%N_2_ reduced mesophile, psychrotroph, and *Pseudomonas* counts in poultry breast fillets. As in the present work Jimenez et al. [39] reported that *Pseudomonas* counts were higher in breast samples packaged in air than in those packaged in 30%CO_2_/70%N_2_. However, these authors reported similar *Pseudomona*s growth in breast packaged in air or under vacuum. These differences could be due to the high permeability of the film used by these authors.

The reduction in *Enterobacterales* counts observed in the chicken legs packaged in 40%CO_2_/60%N_2_ is consistent with the findings of other authors [22,35]. However, Jimenez et al. [39] reported that *Enterobacterales* counts did not reach counts of 6 log cfu/g in breast packaged in 30%Co_2_/70%N_2_ and stored at 4 °C. In the present study *Enterobacterales* counts of 6 log cfu/g were reached on legs packaged in 40%CO_2_/70%N_2_ on day 13 of storage. It should be noted that pH of legs is higher than pH of breasts [36], thus the first ones support better microbial growth. Meredith et al. [22] observed significant *Enterobacterales* reduction after 7 days of storage under 30%CO_2_/60%N_2_. In the present study significant reductions in *Enterobacterales* counts in legs packaged in MAP compared to control were only observed in legs packaged in 40% CO_2_/60%N_2_ after day 1 of storage and only on day 1 on legs packaged in 20% CO_2_/80%N_2_. No significant reduction (*p* > 0.05) in *Enterobacterales* counts in breast packaged in 10% CO_2_ were observed by Meredith et al. [22].

The use of vacuum or atmospheres containing CO_2_ delayed the time to reach mesophiles and psychrotrophic counts of 8 log cfu/g. These results coincide with the findings of other researchers [22,39]. Jimenez et al. [39] detected signs of spoilage in breast packaged in 30%CO_2_/70%N_2_ at day 14 of storage. In the present study chicken legs packaged in 20%CO_2_/80%N_2_ and 40%CO_2_/60%N_2_ remained acceptable until day 10 and 15, respectively. As reported by Jimenez et al. [39] it was observed that the higher the CO_2_ concentration, the longer shelf life for chicken meat. Aerobic spoilage bacteria, mainly *Pseudomonas* are inhibited by concentrations of 20% CO_2_ or greater. A minimum CO_2_ concentration is needed to exhibit and inhibitory effect [20]. The efficacy of packaging chicken in atmospheres containing CO_2_ in extending the shelf life relies on the antimicrobial properties of CO_2_ [20]. Carbon dioxide has antimicrobial properties whereas N_2_ is used as a filling gas replacing O_2_, and is used as an alternative to vacuum packaging.

MAP can affect the survival of *Campylobacter jejuni*. It was observed that *C jejuni* was able to survive better in carbon dioxide atmospheres than in aerobic conditions or vacuum packaging. While MAP is effective at slowing growth of some pathogens, it is not effective against *Campylobacter* spp. These results are consistent with the observation of Beuchat [40] who reported that CO_2_ had a protective effect on *C. jejuni* on chicken meat stored at 5 °C. Additionally, Meredith et al. [22] observed that *Campylobacter* spp. counts were reduced during the storage at 4 °C in all the packaging conditions tested (air or atmosphere containing CO_2_). These authors also reported that the *Campylobacter* decline was faster in poultry samples packaged in air than in those packaged in 30%CO_2_/70%N_2_, although these differences were not statistically significant (*p* > 0.05) with the exception of packaging in 30%CO_2_/70%N_2_ compared to the control samples packaged in air after 17 days of storage. In addition, Boysen et al. [41] observed that *C. jejuni* survived longer in modified atmospheres than in air. Other authors have also reported that MAP is not effective against *Campylobacte*r spp. [42]. These findings could be explained since atmospheres containing CO_2_ inhibit other pathogenic and spoilage bacteria [43]. Moreover, Balamurugan et al. [44] observed that spoilage bacteria present in beef and pork meat are able to use the oxygen making the environment more suitable for *Campylobacter* spp. On the other hand, Hilbert et al. [45] pointed that *C. jejuni* is able to survive under oxygen conditions when cocultured with *Pseudomonas*. Thus, the high microbial load of poultry meat, mainly *Pseudomonas*, could encourage the survival of *C. jejuni* as it is observed in the present study. Producers in the poultry industry have used high concentrations of oxygen to pack fresh poultry (>60%) [46]. Rossaint et al. [46] have studied the effect of atmospheres containing high oxygen (70%CO_2_/30%CO_2_) and high nitrogen (70%N_2_/30%CO_2_) on the spoilage process of poultry breast fillets. These authors observed that the composition of the spoilage bacteria differed between both packaging conditions. However, no differences were observed in mesophilic counts and sensory parameters between both packaging conditions [46]. Enriched O_2_ atmospheres (80% O_2_/20%N_2_) has been reported as effective in reducing *C. jejuni* counts [24]. Meredith et al. [22] reported that *Campylobacter* counts were reduced in poultry packaged in 80% O_2_/20%N_2_, but these packaging conditions also favored the growth of other bacteria on chicken, thus they suggested that the best gaseous mixture for reducing *Campylobacter* counts and extending shelf life was 40%CO_2_/30%O_2_/30%N_2_.

Other authors have also reported the reduction of mesophilic, psychrotrophic, *Pseudomona*s, and *Enterobacterales* counts in poultry meat treated with organic acids and packaged in MAP [47,48,49]. Zeitoun and Debevere [47] studied the combined effect of buffered lactic acid treatment and modified atmosphere packaging (90% CO_2_/10% O_2_) on poultry meat. According to these authors there is a synergistic effect of CO_2_ and lactic acid treatment. Sawaya et al. [48] reported that a combination of vacuum packaging and sorbate treatment reduced microbial counts and extended the shelf-life of poultry carcasses. Jimenez et al. [49] observed that the combined effect of acetic acid treatment and MAP is useful for extending the shelf life of chicken breasts.

Atmospheres containing CO_2_ are often used for packaging chicken since they increase the product shelf-life by decreasing the growth of spoilage bacteria. However, our results suggest that after lactic acid treatment *C. jejuni* survives better under CO_2_ enriched atmospheres than in air. These findings are in agreement with those reported by other authors [23,24]. Smigic et al. [23] studied the survival of *C. jejuni* cells treated with 3% lactic in broth incubated under 80%CO_2_/20%N_2_, air or micro-aerophlilic conditions (10%CO_2_/85%N_2_/5%O_2_) at 4 °C over 7 days. According to these authors *C. jejuni* survives better in CO_2_ enriched atmospheres than in air. Smigdic et al. [23] pointed the importance of combing decontamination with other preservation methods in order to control the growth and survival of foodborne pathogens. Rajkovic et al. [24] reported a reduction of 1.8 log units of *C. jejuni* in chicken legs treated with 10% lactic acid buffered with sodium lactate. However, no additional reductions were observed in chicken packaged in 80%CO_2_/20% N_2_.

## 5. Conclusions

Poultry legs packaged in atmospheres containing 40%CO_2_/60%N_2_, had an extended “sensorial” shelf life. A treatment with 2% lactic acid before packaging in MAP is effective in reducing the initial *C. jejuni* counts and spoilage bacteria, in consequence this combined treatment could improve both the microbial safety and the shelf life of chicken meat.

## Figures and Tables

**Table 1:** Effect of lactic acid on the mesophilic, psychrotrophic, *Pseudomonas, Enterobacterales*, and *Campylobacter jejuni* counts on chicken legs (log cfu/g).

Microbial Group	Treatment	Day of Storage				
		0	1	3	6	8
Mesophiles	Control	5.30 ± 0.30 ^a^	7.08 ± 0.06 ^a^	7.85 ± 0.06 ^a^	8.83 ± 0.02 ^a^	8.84 ± 0.03 ^a^
	1% Lactic acid	3.71 ± 0.28 ^b^	6.41 ± 0.39 ^b^	7.72 ± 0.08 ^a^	7.84 ± 0.09 ^b^	8.06 ± 0.14 ^b^
	2% Lactic acid	3.71 ± 0.28 ^b^	5.88 ± 0.26 ^b^	7.19 ± 0.15 ^b^	7.75 ± 0.04 ^b^	7.87 ± 0.04 ^b^
Psychrotrophs	Control	4.54 ± 0.31 ^a^	6.56 ± 0.09 ^a^	7.65 ± 0.13 ^a^	8.80 ± 0.26 ^a^	8.89 ± 0.03 ^a^
	1% Lactic acid	2.93 ± 0.98 ^b^	5.33 ± 0.32 ^b^	6.92 ± 0.40 ^b^	7.37 ± 0.26 ^b^	7.56 ± 0.13 ^b^
	2% Lactic acid	2.47 ± 0.22 ^b^	5.07 ± 0.19 ^b^	6.34 ± 0.23 ^b^	6.86 ± 0.23 ^c^	6.98 ± 0.18 ^c^
*Pseudomonas*	Control	4.55 ± 0.06 ^a^	5.50 ± 0.17 ^a^	7.17 ± 0.08 ^a^	7.43 ± 0.05 ^a^	7.98 ± 0.04 ^a^
	1% Lactic acid	2.96 ± 0.52 ^b^	5.03 ± 0.04 ^b^	6.14 ± 0.12 ^b^	6.11 ± 0.14 ^b^	7.07 ± 0.24 ^b^
	2% Lactic acid	2.63 ± 0.23 ^b^	4.05 ± 0.54 ^c^	5.41 ± 0.11 ^c^	5.93 ± 0.04 ^b^	6.70 ± 0.06 ^b^
*Enterobacterales*	Control	3.11 ± 0.13 ^a^	5.24 ± 0.18 ^a^	6.39 ± 0.29 ^a^	7.74 ± 0.04 ^a^	7.75 ± 0.03 ^a^
	1% Lactic acid	2.73 ± 0.10 ^b^	4.06 ± 0.25 ^b^	5.79 ± 0.12 ^b^	6.02 ± 0.30 ^b^	6.25 ± 0.21 ^b^
	2% Lactic acid	2.61 ± 0.55 ^b^	3.35 ± 0.38 ^c^	5.60 ± 0.69 ^b^	5.80 ± 0.29 ^b^	6.23 ± 0.25 ^b^
*C. jejuni*	Control	4.75 ± 0.22 ^a^	4.68 ± 0.22 ^a^	4.00 ± 0.06 ^a^	3.84 ± 0.02 ^a^	3.61 ± 0.03 ^a^
	1% Lactic acid	3.65 ± 0.22 ^b^	3.51 ± 0.26 ^b^	3.47 ± 0.07 ^b^	3.74 ± 0.12 ^a^	3.54 ± 0.13 ^a^
	2% Lactic acid	3.33 ± 0.29 ^b^	3.18 ± 0.30 ^b^	3.24 ± 0.23 ^b^	3.21 ± 0.11 ^b^	3.09 ± 0.09 ^b^

Mean ± standard deviation; Mean values in the same column within the same microbial group that are followed by the same letter were not significantly different (*p* > 0.05).

**Table 2:** Overall appearance of chicken legs treated with lactic acid and stored at 4 °C.

Treatment	Day of Storage				
	0	1	3	6	8
Control	7.00 ± 0.00 ^a^	7.00 ± 0.00 ^a^	4.67 ± 0.47 ^a^	2.33 ± 0.47 ^a^	1.11 ± 0.31 ^a^
1% Lactic acid	7.00 ± 0.00 ^a^	7.00 ± 0.00 ^a^	6.11 ± 0.31 ^b^	4.67 ± 0.47 ^b^	2.44 ± 0.50 ^b^
2% Lactic acid	7.00 ± 0.00 ^a^	7.00 ± 0.00 ^a^	6.33 ± 0.47 ^b^	6.11 ± 0.31 ^c^	4.67 ± 0.47 ^c^

Mean ± standard deviation; mean values in the same column that are followed by the same letter were not significantly different (*p* > 0.05).

**Table 3:** Effect of different atmosphere packaging conditions on the mesophilic, psychrotrophic, *Pseudomonas, Enterobacterales*, and *C. jejuni* counts on chicken legs (log cfu/g).

Microbial Group	Batch	Day of Storage							
		0	1	3	6	8	10	13	15
**Mesophiles**	AC	5.15 ± 0.44 ^a^	6.55 ± 0.04 ^a^	7.78 ± 0.04 ^a^	8.95 ± 0.39 ^a^	NI	NI	NI	NI
	AN		5.39 ± 0.13 ^b^	7.01 ± 0.16 ^b^	8.59 ± 0.38 ^a^	9.19 ± 0.15 ^a^	NI	NI	NI
	AV		5.35 ± 0.11 ^b^	6.80 ± 0.13 ^b^	7.53 ± 0.11 ^b^	8.29 ± 0.11 ^b^	8.62 ± 0.12 ^a^	9.00 ± 0.43 ^a^	NI
	AC20		4.89 ± 0.17 ^c^	6.52 ± 0.08 ^c^	7.26 ± 0.17 ^bc^	7.79 ± 0.48 ^bc^	8.10 ± 0.12 ^b^	8.80 ± 0.31 ^a^	NI
	AC40		4.61 ± 0.09 ^c^	6.43 ± 0.04 ^c^	7.00 ± 0.15 ^c^	7.47 ± 0.14 ^c^	7.81 ± 0.11 ^b^	7.85 ± 0.10 ^b^	8.25 ± 0.04
Psychrotophs	AC	4.58 ± 0.43 ^a^	6.62 ± 0.07 ^a^	7.65 ± 0.03 ^a^	8.71 ± 0.43 ^a^	NI	NI	NI	NI
	AN		5.40 ± 0.14 ^b^	6.99 ± 0.14 ^b^	8.49 ± 0.04 ^a^	9.02 ± 0.01 ^a^	NI	NI	NI
	AV		5.38 ± 0.15 ^b^	6.72 ± 0.09 ^b^	7.44 ± 0.08 ^b^	8.00 ± 0.09 ^b^	8.50 ± 0.02 ^a^	8.90 ± 0.21 ^a^	NI
	AC20		4.78 ± 0.22 ^c^	6.50 ± 0.14 ^c^	7.16 ± 0.17 ^bc^	7.64 ± 0.29 ^bc^	8.00 ± 0.05 ^b^	8.70 ± 0.20 ^a^	NI
	AC40		4.50 ± 0.03 ^c^	6.41 ± 0.30 ^c^	6.90 ± 0.20 ^c^	7.35 ± 0.02 ^c^	7.75 ± 0.05 ^b^	7.78 ± 0.11 ^b^	8.00 ± 0.12
*Pseudomonas*	AC	4.34 ± 0.14 ^a^	6.58 ± 0.04 ^a^	7.70 ± 0.11 ^a^	8.60 ± 0.39 ^a^	NI	NI	NI	NI
	AN		5.40 ± 0.14 ^b^	6.80 ± 0.14 ^b^	8.29 ± 0.16 ^a^	8.84 ± 0.12 ^a^	NI	NI	NI
	AV		5.35 ± 0.04 ^b^	6.74 ± 0.17 ^b^	7.40 ± 0.15 ^b^	8.00 ± 0.09 ^b^	8.43 ± 0.15 ^a^	8.70 ± 0.02 ^a^	NI
	AC20		4.88 ± 0.05 ^c^	6.46 ± 0.23 ^b^	7.02 ± 0.30 ^b^	7.52 ± 0.40 ^bc^	7.90 ± 0.06 ^b^	8.60 ± 0.06 ^a^	NI
	AC40		4.42 ± 0.12 ^c^	6.35 ± 0.04 ^c^	6.81 ± 0.18 ^c^	7.30 ± 0.08 ^c^	7.53 ± 0.44 ^b^	7.71 ± 0.07 ^b^	7.98 ± 0.05
*Enterobacterales*	AC	2.68 ± 0.19 ^a^	4.67 ± 0.07 ^a^	5.54 ± 0.35 ^a^	6.25 ± 0.32 ^a^	NI	NI	NI	NI
	AN		4.24 ± 0.48 ^a^	5.40 ± 0.38 ^a^	6.19 ± 0.16 ^a^	6.00 ± 0.12 ^a^	NI	NI	NI
	AV		4.38 ± 0.36 ^a^	5.51 ± 0.14 ^a^	6.09 ± 0.15 ^a^	6.31 ± 0.11 ^a^	6.57 ± 0.30 ^a^	6.72 ± 0.13 ^a^	NI
	AC20		3.63 ± 0.14 ^b^	5.46 ± 0.12 ^a^	6.18 ± 0.18 ^a^	6.30 ± 0.38 ^a^	6.36 ± 0.11 ^a^	6.58 ± 0.02 ^a^	NI
	AC40		3.43 ± 0.11 ^b^	4.99 ± 0.19 ^b^	5.38 ± 0.17 ^b^	5.43 ± 0.14 ^b^	5.80 ± 0.30 ^b^	6.35 ± 0.09 ^a^	7.09 ± 0.30
*C. jejuni*	AC	4.59 ± 0.15 ^a^	4.44 ± 0.07 ^a^	4.00 ± 0.13 ^a^	3.79 ± 0.09 ^a^	NI	NI	NI	NI
	AN		4.53 ± 0.08 ^a^	4.41 ± 0.03 ^b^	4.33 ± 0.21 ^b^	4.34 ± 0.12 ^a^	NI	NI	NI
	AV		4.49 ± 0.12 ^a^	4.41 ± 0.17 ^b^	4.37 ± 0.18 ^b^	4.38 ± 0.34 ^a^	4.28 ± 0.16 ^a^	4.18 ± 0.15 ^a^	NI
	AC20		4.52 ± 0.18 ^a^	4.50 ± 0.22 ^b^	4.49 ± 0.17 ^b^	4.45 ± 0.14 ^a^	4.41 ± 0.09 ^a^	4.20 ± 0.17 ^a^	NI
	AC40		4.70 ± 0.08 ^a^	4.53 ± 0.04 ^b^	4.57 ± 0.18 ^b^	4.55 ± 0.09 ^a^	4.42 ± 0.37 ^a^	4.30 ± 0.12 ^a^	4.21 ± 0.12

Mean ± standard deviation; mean values in the same column within the same microbial group that are followed by the same letter were not significantly different (*p* > 0.05). NI, not investigated; treatment conditions: Batch AC: packaging in air; Batch AN: packaging in 100%N_2_; Batch AV: packaging in vacuum; Batch AC20: packaging in 20%CO_2_/80%N_2_; Batch AC40: packaging in 40%CO_2_/60%N_2_.

**Table 4:** Overall appearance of chicken legs packaged under different conditions and stored at 4 °C.

Batch	Day of Storage							
	0	1	3	6	8	10	13	15
AC	7.00 ± 0.00 ^a^	6.67 ± 0.47 ^a^	4.11 ± 0.31 ^a^	2.11 ± 0.31 ^a^	NI	NI	NI	NI
AN	7.00 ± 0.00 ^a^	7.00 ± 0.00 ^a^	5.67 ± 0.47 ^b^	4.33 ± 0.47 ^b^	2.67 ± 0.47 ^a^	NI	NI	NI
AV	7.00 ± 0.00 ^a^	7.00 ± 0.00 ^a^	6.11 ± 0.31 ^b^	4.67 ± 0.47 ^b^	4.11 ± 0.31 ^b^	3.67 ± 0.47 ^a^	2.33 ± 0.67 ^a^	NI
AC20	7.00 ± 0.00 ^a^	7.00 ± 0.00 ^a^	6.67 ± 0.47 ^b^	6.11 ± 0.31^c^	5.67 ± 0.47^c^	4.11 ± 0.31 ^a^	2.67 ± 0.47 ^a^	NI
AC40	7.00 ± 0.00 ^a^	7.00 ± 0.00 ^a^	6.33 ± 047 ^b^	6.33 ± 0.47^c^	6.33 ± 0.47^c^	5.67 ± 0.47 ^b^	4.11 ± 0.31 ^b^	2.67 ± 0.47

Mean ± standard deviation; mean values in the same column that are followed by the same letter were not significantly different (*p* > 0.05). NI, not investigated; treatment conditions: Batch AC: packaging in air; Batch AN: packaging in 100%N_2_; Batch AV: packaging in vacuum; Batch AC20: packaging in 20%CO_2_/80%N_2_; Batch AC40: packaging in 40%CO_2_/60%N_2_.

**Table 5:** Effect of 2% lactic acid on the mesophilic, psychrotrophic, *Pseudomonas, Enterobacterales*, and *C. jejuni* counts on chicken legs packaged under different modified atmospheres (log cfu/g).

Microbial Group	Batch	Day of Storage								
		0	1	3	6	8	10	13	15	17
Meosphiles	BC	4.71 ± 0.02 ^a^	6.25 ± 0.15 ^a^	7.20 ± 0.13 ^a^	8.72 ± 0.10 ^a^	NI	NI	NI	NI	NI
	BA	3.90 ± 0.15 ^b^	4.90 ± 0.03 ^b^	6.28 ± 0.41 ^b^	6.83 ± 0.15 ^b^	7.91 ± 0.10 ^a^	8.14 ± 0.16 ^a^	8.84 ± 0.10 ^a^	NI	NI
	BV	3.90 ± 0.15 ^b^	4.76 ± 0.39 ^b^	5.87 ± 0.39 ^b^	6.11 ± 0.29 ^c^	6.16 ± 0.05 ^b^	6.24 ± 0.15 ^b^	7.54 ± 0.06 ^b^	8.07 ± 0.31 ^a^	8.57 ± 0.12 ^a^
	BC20	3.90 ± 0.15 ^b^	3.85 ± 0.21 ^c^	4.19 ± 0.53 ^c^	5.34 ± 0.11^d^	5.52 ± 0.11 ^c^	5.67 ± 0.18 ^c^	6.89 ± 0.13 ^c^	7.85 ± 0.45 ^a^	8.48 ± 0.33 ^a^
	BC40	3.90 ± 0.15 ^b^	3.73 ± 0.07 ^c^	3.86 ± 0.05 ^c^	4.89 ± 0.30^d^	4.95 ± 0.13 ^d^	5.18 ± 0.08^d^	5.95 ± 0.08^d^	6.95 ± 0.11 ^b^	7.93 ± 0.11 ^b^
Psychrotophs	BC	4.32 ± 0.08 ^a^	6.16 ± 0.43 ^a^	7.39 ± 0.11 ^a^	8.98 ± 0.02 ^a^	NI	NI	NI	NI	NI
	BA	2.30 ± 0.63 ^b^	4.88 ± 0.24 ^b^	5.95 ± 0.41 ^b^	6.83 ± 0.15 ^b^	6.76 ± 0.02 ^a^	8.13 ± 0.35 ^a^	8.75 ± 0.20 ^a^	NI	NI
	BV	2.30 ± 0.63 ^b^	4.65 ± 0.32 ^b^	5.59 ± 0.16 ^b^	6.23 ± 0.08 ^c^	5.86 ± 0.12 ^b^	6.18 ± 0.51 ^b^	7.78 ± 0.13 ^b^	8.35 ± 0.44 ^a^	8.40 ± 0.12 ^a^
	BC20	2.30 ± 0.63 ^b^	3.44 ± 0.29 ^c^	4.14 ± 0.63 ^c^	5.24 ± 0.22 ^c^	5.30 ± 0.45 ^b^	5.74 ± 0.20 ^b^	6.87 ± 0.10 ^c^	7.82 ± 0.29 ^b^	8.18 ± 0.08 ^a^
	BC40	2.30 ± 0.63 ^b^	3.10 ± 0.14 ^c^	3.80 ± 0.34 ^c^	4.24 ± 0.32^d^	4.59 ± 0.28 ^c^	5.16 ± 0.11 ^c^	5.65 ± 0.32^d^	6.57 ± 0.36 ^c^	7.80 ± 0.17 ^b^
*Pseudomonas*	BC	4.31 ± 0.06 ^a^	5.73 ± 0.08 ^a^	7.00 ± 0.41 ^a^	7.47 ± 0.28 ^a^	NI	NI	NI	NI	NI
	BA	2.33 ± 0.14 ^b^	4.75 ± 0.54 ^b^	5.33 ± 0.51 ^b^	5.72 ± 0.10 ^b^	6.98 ± 0.02 ^a^	8.16 ± 0.32 ^a^	8.60 ± 0.10 ^a^	NI	NI
	BV	2.33 ± 0.14 ^b^	4.26 ± 0.30 ^b^	4.95 ± 0.17 ^b^	5.16 ± 0.38 ^c^	5.26 ± 0.26 ^b^	6.07 ± 0.15 ^b^	7.82 ± 0.16 ^b^	8.21 ± 0.30 ^a^	8.44 ± 0.38 ^a^
	BC20	2.33 ± 0.14 ^b^	3.22 ± 0.24 ^c^	3.72 ± 0.15 ^c^	4.91 ± 0.04 ^c^	5.19 ± 0.49 ^b^	5.75 ± 0.18 ^b^	6.70 ± 0.39 ^c^	7.80 ± 0.13 ^a^	8.29 ± 0.43 ^a^
	BC40	2.33 ± 0.14 ^b^	3.00 ± 0.10 ^c^	3.34 ± 0.22 ^c^	4.15 ± 0.32^d^	4.38 ± 0.15 ^c^	5.05 ± 0.39 ^c^	5.30 ± 0.23^d^	6.45 ± 0.39 a	7.40 ± 0.23 ^b^
*Enterobacterales*	BC	2.76 ± 0.33 ^a^	4.49 ± 038 ^a^	5.90 ± 0.32 ^a^	7.86 ± 0.06 ^a^	NI	NI	NI	NI	NI
	BA	2.06 ± 0.15 ^b^	2.24 ± 0.18 ^b^	4.21 ± 0.11 ^b^	5.91 ± 0.12 ^b^	6.13 ± 0.05 ^a^	6.58 ± 0.23 ^a^	7.00 ± 0.06 ^a^	NI	NI
	BV	2.06 ± 0.15 ^b^	2.00 ± 0.15 ^b^	3.83 ± 0.08 ^c^	4.96 ± 0.07 ^c^	5.26 ± 0.26 ^b^	5.65 ± 0.12 ^b^	6.82 ± 0.04 ^b^	6.45 ± 0.14 ^a^	6.60 ± 0.36 ^a^
	BC20	2.06 ± 0.15 ^b^	2.18 ± 0.35 ^b^	3.55 ± 0.59 ^cd^	3.86 ± 0.24^d^	5.03 ± 0.09 ^b^	5.04 ± 0.24 ^c^	6.00 ± 0.09 ^b^	6.40 ± 0.16 ^a^	6.45 ± 0.17 ^a^
	BC40	2.06 ± 0.15 ^b^	2.14 ± 0.34 ^b^	2.94 ± 0.17^d^	3.26 ± 0.29^d^	4.82 ± 0.18 ^b^	4.96 ± 0.39 ^c^	5.50 ± 0.14 ^c^	5.93 ± 0.13 ^b^	5.94 ± 0.17 ^b^
*C. jejuni*	BC	4.88 ± 0.06 ^a^	4.61 ± 0.05 ^a^	4.41 ± 0.09 ^a^	3.85 ± 0.07 ^a^	NI	NI	NI	NI	NI
	BA	3.33 ± 0.12 ^b^	3.01 ± 0.03 ^b^	2.81 ± 0.33 ^b^	2.70 ± 0.27 ^b^	2.78 ± 0.11 ^a^	2.70 ± 0.10 ^a^	2.30 ± 0.15 ^a^	NI	NI
	BV	3.33 ± 0.12 ^b^	3.20 ± 0.13 ^b^	2.84 ± 0.39 ^b^	2.90 ± 0.06 ^b^	2.90 ± 0.32 ^a^	2.71 ± 0.16 ^a^	2.77 ± 0.13 ^ab^	2.68 ± 0.20 ^a^	2.75 ± 0.25 ^a^
	BC20	3.33 ± 0.12 ^b^	3.25 ± 0.13 ^b^	2.90 ± 0.19 ^b^	2.98 ± 0.15 ^b^	2.95 ± 0.39 ^a^	2.91 ± 0.09 ^a^	3.06 ± 0.49 ^b^	2.84 ± 0.12 ^a^	3.13 ± 0.18 ^a^
	BC40	3.33 ± 0.12 ^b^	3.49 ± 0.23 ^b^	3.00 ± 0.13 ^b^	3.10 ± 0.02 ^b^	2.98 ± 0.13 ^a^	3.08 ± 0.06 ^a^	3.14 ± 0.12 ^b^	2.87 ± 0.12 ^a^	3.13 ± 0.18 ^a^

Mean ± standard deviation; mean values in the same column within the same microbial group that are followed by the same letter were not significantly different (*p* > 0.05). NI, not investigated; treatment conditions: Batch BC: control without lactic acid treatment, packaging in air; Batch BA: 2% lactic acid, packaging in air; Batch BV: 2% lactic acid, packaging in vacuum; Batch BC20: 2% lactic acid, packaging in 20%CO_2_/80%N_2_; Batch BC40: 2% lactic acid, packaging in 40%CO_2_/60%N_2_.

**Table 6:** Overall appearance of chicken legs treated with 2% lactic acid and packaged under different conditions.

Batch	Day of Storage								
	0	1	3	6	8	10	13	15	17
BC	7.00 ± 0.00 ^a^	6.67 ± 0.47 ^a^	4.33 ± 0.47 ^a^	2.11 ± 0.31 ^a^	NI	NI	NI	NI	NI
BA	7.00 ± 0.00 ^a^	7.00 ± 0.00 ^a^	6.33 ± 0.47 ^b^	6.11 ± 0.31 ^b^	4.67 ± 0.47 ^a^	4.11 ± 0.31 ^a^	2.44 ± 0.50 ^a^	NI	NI
BV	7.00 ± 0.00 ^a^	7.00 ± 0.00 ^a^	6.67 ± 0.47 ^b^	6.44 ± 0.50 ^b^	6.33 ± 0.47 ^b^	5.67 ± 0.47 ^b^	5.11 ± 0.31 ^b^	3.44 ± 0.50 ^a^	2.11 ± 0.31 ^a^
BC20	7.00 ± 0.00 ^a^	7.00 ± 0.00 ^a^	6.67 ± 0.47 ^b^	6.67±0.47 ^b^	6.44 ± 0.50 ^b^	6.11 ± 0.31 ^b^	5.67 ± 0.47 ^b^	3.67 ± 0.47 ^a^	2.44 ± 0.50 ^a^
BC40	7.00 ± 0.00 ^a^	7.00 ± 0.00 ^a^	6.67 ± 0.47 ^b^	6.67±0.47 ^b^	6.44 ± 0.50 ^b^	6.11 ± 0.31 ^b^	5.67 ± 0.47 ^b^	4.11 ± 0.31 ^a^	3.67 ± 0.47 ^b^

Mean ± standard deviation; mean values in the same column that are followed by the same letter were not significantly different (*p* > 0.05). NI, not investigated. treatment conditions: Batch BC: control without lactic acid treatment, packaging in air; Batch BA: 2% lactic acid, packaging in air; Batch BV: 2% lactic acid, packaging in vacuum; Batch BC20: 2% lactic acid, packaging in 20%CO_2_/80%N_2_; Batch BC40: 2% lactic acid, packaging in 40%CO_2_/60%N_2_.

**Table 7:** Efficacy of lactic acid treatment on the decontamination of chicken.

Microorganism	Product	Lacic Acid (%)	Reduction log (CFU)	Exposure Time	Sampling Time	Storage Temperature (°C)	Reference
Psychrotophs	Legs	3%	0.79	5 min	After treatment	4	[28]
Psychrotophs	Breasts	3%	0.9	5 min	After treatment	4	[28]
Mesophiles	Carcasses	2%	2.54	10 min	Day 8	2	[29]
Psychrotophs	Carcasses	2%	2.21	10 min	Day 8	2	[29]
*Enterobacterales*	carcasses	2%	2.65	10 min	Day 8	2	[29]
Mesophiles	Breasts	1–2%	0.53–2.36	10–30 min	After treatment	4	[30]
*Pseudomonas*	Breasts	1%	0.89	10 min	After treatment	4	[30]
*Pseudomonas*	Breasts	2%	1.18	10 min	After treatment	4	[30]
*Pseudomonas*	Breasts	1%	2.44	10 min	Day 7	4	[30]
*Pseudomonas*	Breasts	2%	2.23	10 min	Day 7	4	[30]
Mesophiles	Carcasses	1%	0.3	10 s	After treatment	0	[31]
Mesophiles	Carcasses	2%	0.6	10 s	After treatment	0	[31]
Psychrotophs	Carcasses	1%	0.7	10 s	After treatment	0	[31]
Psychrotophs	Carcasses	2%	1	10 s	After treatment	0	[31]
*C. jejuni*	Legs	1%	0.36	10 min	After treatment	4	[19]
*C. jejuni*	Breasts	1%	1.27	10 min	After treatment	4	[19]
*C. jejuni*	Legs	3%	1.06	10 min	After treatment	4	[19]
*C. jejuni*	Breasts	3%	1.98	10 min	After treatment	4	[19]
*C. jejuni*	Legs	1%	0.05	10 min	Day 7	4	[19]
*C. jejuni*	Breasts	1%	0.81	10 min	Day 7	4	[19]
*C. jejuni*	Legs	3%	0.45	10 min	Day 7	4	[19]
*C. jejuni*	Breasts	3%	1.15	10 min	Day 7	4	[19]
*Campylobacter* spp.	Chicken skin	1%	0.63	15 s	After treatment	4	[32]
*Campylobacter* spp.	Chicken skin	3%	1.12	15 s	After treatment	4	[32]
*Campylobacter* spp.	Chicken skin	1%	0.68	15 s	Day 5	4	[32]
*Campylobacter* spp.	Chicken skin	3%	2.33	15 s	Day 5	4	[32]
*C. jejuni*	Chicken skin	2.5%	0.74	1 min	After treatment	5	[34]
*C. jejuni*	Breast fillets	2.5%	0.4	1 min	After treatment	5	[34]
*C. jejuni*	Breast fillets	2.5%	1.5	1 min	Day 1	5	[34]
*C. jejuni*	Wings	5%	2.3	30 s	After treatment	5	[35]

## References

[1] EFSA (2018). The European Union summary report on trends and sources of zoonoses, zoonotic agents and food-borne outbreaks in 2017. EFSA J..

[2] EFSA (2010). Analysis of the baseline survey on the prevalence of *Campylobacter* in broiler batches and of *Campylobacter* and *Salmonella* on broiler carcasses in the EU, 2008—Part A: *Campylobacter* and *Salmonella* prevalence estimates. EFSA J..

[3] Perez-Arnedo I., Gonzalez-Fandos E. (2019). Prevalence of *Campylobacter* spp. in poultry in three Spanish farms, a slaughterhouse and a further processing plant. Foods.

[4] Wingstrand A., Neimann J., Engberg J., Nielsen E.M., Gerenr-Smidt P., Wegener H.C., Molbak L. (2006). Fresh chicken as main risk factor for campylobacteriosis, Denmark. Emerg. Infect. Dis..

[5] Rosenquist H., Nielsen N.L., Sommer H.M., Norrung B., Christensen B.B. (2003). Quantitative risk assessment of human campylobacteriosis associated with thermophilic *Campylobacter* species in chickens. Int. J. Food Microbiol..

[6] Surekha M., Reddy S.M., Robinson R.K., Batt C.A., Patel C. (2000). Preservatives. Classsification and properties. Encyclopedia of Food Microbiology.

[7] González-Fandos E., Maya N., Pérez-Arnedo I. (2015). Effect of propionic acid on *Campylobacter jejuni* attached to chicken skin during refrigerated storage. Int. Microbiol..

[8] González-Fandos E., Maya N. (2016). Effect of malic acid on *Campylobacter jejuni* attached to chicken skin during refrigerated storage. J. Food Process. Pre..

[9] Smulders F.J.M., Greer G.G. (1998). Integrating microbial decontamination with organic acids in HACCP programmes for muscle foods: Prospects and controversies. Int. J. Food Microbiol..

[10] Dickson J.S., Anderson M.E. (1992). Microbiological decontamination of food animal carcasses by washing and sanitizing systems: A review. J. Food Prot..

[11] Official Journal of the European Union (2013). Commission regulation (EU) No 101/2013 of 4 February 2013, concerning the use of lactic acid to reduce microbiological surface contamination on bovine carcasses. Off. J. Eur. Union.

[12] EFSA (2018). Evaluation of the safety and efficacy of the organic acids lactic and acetic acids to reduce microbiological surface contamination on pork carcasses and pork cuts. EFSA J..

[13] Li Y., Slavik M.F., Walker J.T., Xiong H. (1997). Pre-Chill Spray of Chicken Carcasses to Reduce Salmonella typhimurium. J. Food Sci..

[14] González-Fandos E., Domínguez J.L. (2006). Efficacy of lactic acid against *Listeria monocytogenes* attached to poultry skin during refrigerated storage. J. Appl. Microbiol..

[15] Baserisali M., Bahador N., Kapadnis B.P. (2006). 2006 Effect of heat and food preservatives on survival of thermophilic *Campylobacter* isolates in food products. Res. J. Microbiol..

[16] Zhao T., Doyle M.P. (2006). Reduction of *Campylobacter jejuni* on chicken wings by chemical treatments. J. Food Protect..

[17] Birk T., Gronlund A.C., Christensen B.B., Knochel S., Lohse K., Rosenquist H. (2010). Effect of organic acids and marination ingredients on the survival of *Campylobacter jejuni* on meat. J. Food Protect..

[18] Cudjoe K.S., Kapperud G. (1991). The effect of lactic acid sprays on *Campylobacter jejuni* inoculated onto poultry carcasses. Acta Vet. Scand..

[19] Coşansu S., Ayhan K. (2010). Effects of lactic and acetic acid treatments on *Campylobacter jejuni* inoculated onto chicken leg and breast meat during storage at 4 °C and −18 °C. J. Food Process. Pres..

[20] Arvanitoyannis I.S., Stratakos A. (2012). Application of modified atmosphere packaging and active/smart technologies to red meat and poultry: A review. Food Bioprocess Technol..

[21] Macé S., Haddad N., Zagorec M., Tresse O. (2016). Influence of measurement and control of microaerobic gaseous atmospheres in methods for *Campylobacter* growth studies. Food Microbiol..

[22] Meredith H., Valdramidis V., Rotabakk B.T., Sivertsvik M., McDowell D., Bolton D.J. (2014). Effect of different modified atmospheric packaging (MAP) gaseous combinations on *Campylobacter* and the shelf-life of chilled poultry fillets. Food Microbiol..

[23] Smigic N., Rajkovic A., Nielsen D.S., Arneborg N., Siegumfeldt H., Devlieghere F. (2010). Survival of lactic acid and chlorine dioxide treated *Campylobacter jejuni* under suboptimal conditions of pH, temperature and modified atmosphere. Int. J. Food Microbiol..

[24] Rajkovic A., Tomic N., Smigic N., Uyttendaele M., Ragaert P., Devlieghere F. (2010). Survival of *Campylobacter jejuni* on raw chicken legs packed in high-oxygen or highcarbon dioxide atmosphere after the decontamination with lactic acid/sodium lactate buffer. Int. J. Food Microbiol..

[25] Gonzalez-Fandos E., Herrera B. (2014). Efficacy of Acetic Acid against *Listeria monocytogenes* Attached to Poultry Skin during Refrigerated Storage. Foods.

[26] Adelou M., Alnajar S., Naushad S., Gupta R.S. (2016). Genome-based phylogeny and taxonomy of the ‘*Enterobacteriales*’: Proposal for *Enterobacterales* ord. nov. divided into the families *Enterobacteriaceae*, *Erwiniaceae* fam. nov., *Pectobacteriaceae* fam. nov., *Yersiniaceae* fam. nov., *Hafniaceae* fam. nov., *Morganellaceae* fam. nov., and *Budviciaceae* fam. nov.. Int. J. Systematic Evol. Microbiol..

[27] ISO (2006). Microbiology of Food and Animal Feeding Stuffs. Horizontal Method for the Detection and Enumeration of Campylobacter spp. —Part 2: Colony Count Technique.

[28] Kolsarici N., Candogan K. (1995). The effects of potassium sorbate and lactic acid on the shelf life of vacuum-packaged chicken meats. Poultry Sci..

[29] Morshedy A.E.M.A., Sallam K.I. (2009). Improving the microbial quality and shelf life of chicken carcasses by trisodium phosphate and lactic acid dipping. Int. J. Poultry Sci..

[30] Anang D.M., Rusul G., Hoo Ling F., Bhat R. (2010). Inhibitory effects of lactic acid and lauricidin on spoilage organisms of chicken breast during storage at chilled temperature. Int. J. Food Microbiol..

[31] Van der Marel G.M., Van Logtestijn J.G., Mossel D.A. (1988). Bacteriological quality of broiler carcasses as affected by in-plant lactic acid decontamination. Int. J. Food Microbiol..

[32] Meredith H., Walsh D., McDowell D.A., Bolton D.J. (2013). An investigation of the immediate and storage effects of chemical treatments on Campylobacter and sensory characteristics of poultry meat. Int. J. Food Microbiol..

[33] Bell C., Kyriakides A. (2009). Campylobacter: A Practical Approach to the Organism and Its Control.

[34] Riedel T.C., BrØndsted L., Rosenquist H., Haxgart H.S., Christensen B.B. (2009). Chemical decontamination of *Campylobacter jejuni* on chicken skin and meat. J. Food Protect..

[35] Shen C., Lemonakisa L., Etienneb X., Lia K., Jianga W., Adlerc J.M. (2019). Evaluation of commercial antimicrobials against stress-adapted *Campylobacter jejuni* on broiler wings by using immersion and electrostatic spray and an economic feasibility analysis. Food Control.

[36] Barnes E. (1976). Microbiological problems of poultry at refrigerator temperatures—A review. J. Sci. Food Agric..

[37] Cherrington C.A., Hinton M., Mead G.C., Chopra I. (1991). Organic acids: Chemistry, antibacterial activity and practical applicactions. Adv. Microb. Physiol..

[38] Stanolevic-Nikolic S., Dimic G., Mojovic L., Pejin J., Djukic-Vukovic A., Kocic-Tanackov S. (2016). Antimicrobial activity of lactic acid against pathogen and spoilage microorganisms. J. Food Process. Preserv..

[39] Jiménez S.M., Salsi M.S., Tiburzi M.C., Rafaghelli R.C., Tessi M.A., Coutaz V.R. (1997). Spoilage microflora in fresh chicken breast stored at 4 °C: Influence of packaging methods. J. Appl. Microbiol..

[40] Beuchat L.R. (1985). Efficacy of media and methods for detecting and enumeration *C. jejuni* in refrigerated chicken meat. Appl. Env. Microbiol..

[41] Boysen L., Knøchel S., Rosenquist H. (2007). Survival of *Campylobacter jejuni* in different gas mixtures. FEMS Microbiol. Lett..

[42] Baer A.A., Miller J.J., Dieger A.C. (2013). Pathogens of interest to the pork industry: A review of research on interventions to assure food safety. Comprenhesive Rev. Food Sci. Food Saf..

[43] Huat J.T.Y., Aziz S.A., Abu J., Ghazala F.M., Cliilek T.Z.T., Ahmaan N., Sandra A., Nishibuchi M., Radu S. (2010). Thermophilic *Campylobacter* spp occurrence on chicken at farm, slaughterhouse and retail. Int. J. Poult. Sci..

[44] Balamurugan S., Nattress F.M., Baker L.P., Dilts B.D. (2011). Survival of *Campylobacter jejuni* on beef and pork under vacuum packaged and retail storage conditions: Examination of the role of natural meat microflora on *Campylobacter jejuni survival*. Food Microbiol..

[45] Hilbert F., Scherwittzel M., Paulsen P., Szostak M.P. (2010). Survival of *Campylobacter jejuni* under conditions of atmospheric oxygen tension with the support of *Pseudomonas* spp.. Appl. Environ..

[46] Rossaint S., Klausmann S., Kreyenschmidt J. (2015). Effect of high-oxygen and oxygen-free modified atmosphere packaging on the spoilage process of poultry breast fillets. Poult. Sc..

[47] Zeitoun A.A.M., Debevere J.M. (1992). Decontamination with lactic acid/sodium lactate buffer in combination with modified atmosphere packaging effects on the shelf life of fresh poultry. Int. J. Food Microbiol..

[48] Sawaya W.N., Elnawawy A.S., Al-Zenki S., Al-Otaibi J., Al-Omirah H., Al-Amiri H. (1995). Storage stability of chicken as affected by map and lactic acid treatment. J. Food Sci..

[49] Jiménez S.M., Salsi M.S., Tiburzi M.C., Rafaghelli R.C., Pirovani M.E. (1999). Combined use of acetic acid treatment and modified atmosphere packaging for extending the shelf-life of chilled chicken breast portions. J. Appl. Microbiol..

